# Changing organizational culture: using the CEO cancer gold standard policy initiatives to promote health and wellness at a school of public health

**DOI:** 10.1186/s12889-015-2186-3

**Published:** 2015-09-03

**Authors:** Samuel D. Towne, Kelsey E. Anderson, Matthew Lee Smith, Deborah Vollmer Dahlke, Debra Kellstedt, Ninfa Pena Purcell, Marcia G. Ory

**Affiliations:** Department of Health Promotion and Community Health Sciences, Texas A&M Health Science Center School of Public Health, 1266 TAMU, College Station, TX 77843-1266 USA; Department of Health Policy and Management, Texas A&M Health Science Center School of Public Health, College Station, TX 77843-1266 USA; Department of Health Promotion and Behavior, College of Public Health, The University of Georgia, Athens, GA 30602 USA; Texas A&M AgriLife Extension Service, Texas A&M University, College Station, TX 77843-1266 USA

## Abstract

**Background:**

Worksite wellness initiatives for health promotion and health education have demonstrated effectiveness in improving employee health and wellness. We examined the effects of a multifaceted health promotion campaign on organizational capacity to meet requirements to become CEO Cancer Gold Standard Accredited.

**Methods:**

We conducted an online survey to assess perceived organizational values and support for the five CEO Cancer Gold Standard Pillars for cancer prevention: tobacco cessation; physical activity; nutrition; cancer screening and early detection; and accessing information on cancer clinical trials. Baseline and follow-up surveys were sent 6-months apart to faculty, staff, and students at a school of public health to test the impact of a multifaceted health promotion campaign on perceived organizational change. Descriptive analyses were used to characterize percent improvement. Multivariate logistic regression analyses were used to control for participants’ university status.

**Results:**

The current organizational culture highly supported tobacco cessation at both time points. Significant improvements (*p* < .05) from baseline to follow-up were observed for questions measuring organizational values for ‘prevention, screening, and early detection of cancer’ and ‘accessing cancer treatment and clinical trials’.

**Conclusions:**

Health promotion and education efforts using multiple approaches were effective to improve perceived organizational values and support for cancer prevention and early detection, and increase access to information about cancer clinical trials. Future studies are needed to examine broader impacts of implementing worksite health promotion initiatives.

## Background

Worksites provide an opportunity to intervene in employees’ health, especially to ameliorate poor lifestyle choices associated with an increased risk for chronic diseases such as cancer. Lifestyle and behavioral factors, such as tobacco use or alcohol consumption throughout the life course, increase an individual’s risk of developing several cancers [1–4]. Obesity, diet, and physical activity are also interrelated risk factors for many common cancers [5–7]. Conversely, health enhancing behaviors such as a balanced, healthy diet, and participation in regular physical activity have been linked to lower risk of several cancers [8–12].

Several settings have been utilized to raise awareness about health and wellness strategies, especially cancer prevention and control topics. The worksite offers opportunities to intervene in many cancer-related health behaviors and lifestyle choices [13, 14]. Worksite health promotion strategies promote organizational success in several ways (e.g. leadership engagement) [15]. This is especially true for the prevention of cancer, where organizations can benefit both directly through reduced medical costs associated with treatments and benefits, and indirectly through lower absenteeism, increased productivity, and reduced disability or workers’ compensation claims [16]. Thus, an employer’s investment in a healthy workforce is critical to reduce the costs associated with cancer and other potentially preventable diseases and conditions [13].

The CEO Cancer Gold Standard accreditation, an initiative from the CEO Roundtable on Cancer that grew from C-Change (a nonprofit organization with representatives from a variety of organizations including the National Cancer Institute), is a worksite wellness initiative focusing on cancer prevention and quality health care for cancer survivors [17]. The CEO Cancer Gold Standard focuses on five key strategies, known as the Five Pillars, which include: prevention (tobacco-free workplace, nutrition, physical activity, healthy weight, vaccines); screening; cancer clinical trials; quality treatment and survivorship; and health education and health promotion [18]. These strategies are associated with specific recommendations that can be applied to existing employee wellness activities and policies, or utilized to formulate novel employee wellness campaigns addressing multiple levels within an organization. In addition to education about tobacco, nutrition, and physical activity, the CEO Cancer Gold Standard includes strategies to educate employees and their families about other cancer risk factors, and to promote healthy behaviors that can decrease cancer risk, such as maintaining a healthy weight and receiving recommended vaccinations. The Five Pillars also encourage employers to provide insurance coverage for evidence-based cancer screenings, facilitate clinical trial access, and enable employees to access quality treatments [18]. Screenings can reduce costs to employers by facilitating the early detection of cancers [19, 20]. Reducing cost barriers to screening can encourage participation [19, 21–23]. The CEO Round Table on Cancer’s recommendation to ensure health benefit plans reduce barriers to cancer clinical trials is based in prior research [16]. Because cancer survivors and their caregivers face unique health challenges, the Five Pillars encourage employers to meet the needs of these individuals in several ways (e.g. health insurance benefits that cannot be eliminated during or after participation in cancer clinical trials) [18].

Organizational capacity is central to achieving CEO Cancer Gold Standard accreditation. Disseminating and implementing innovations (e.g. promoting health promotion efforts) in organizations requires an appreciation for individual (knowledge, beliefs) and organizational (e.g. culture, implementation climate) characteristics [24]. Thus, organizations should be aware of these items if they are to successfully implement and disseminate innovative policies. Given the CEO Cancer Gold Standard strategy targets broader change, the Ecological Model provides a framework to plan health programs that can help address the Five Pillars [25]. This model represents a population-based approach to improving health by affecting the larger environment in which people work.

As recognized in the work of Kwon et al. [26], an effective wellness program must consider both individual behavior change in addition to a supportive organizational culture. To promote change across sectors of the worksite, health promotion programs should ideally focus on the individual, interpersonal, organization/community, and policy levels. When adopting evidenced-base practices that address these areas, worksites have the potential to influence employee health in general, and cancer prevention specifically. Theory-driven wellness programs that recognize the importance of organizational supports are critical for achieving long-term and sustainable impact [27–29]. The CEO Gold Standard accreditation process is an example of an organizational approach to initiating and sustaining both employer and employee health behaviors.

A variety of firms and organizations have sought CEO Cancer Gold Standard accreditation (http://www.cancergoldstandard.org/accredited-organizations). There are now over 175 accredited programs from a variety of academic, nonprofit, and business organizations, many of these are large organizations. Johnson & Johnson, one of the first organizations to become accredited, did so by modifying an existing employee health and wellness program [30]. As the first university to become accredited in 2008, the University of North Dakota also used the CEO Cancer Gold Standard to promote worksite wellness [31]. A few academic health centers such as The University of Colorado Health, The University of Texas Southwestern Medical Center, and Vanderbilt University Medical Center have also been accredited. One of the major elements of CEO accreditation is the creation of a tobacco-free workplace. Less is known about other ways CEO accreditation changes organizational culture in ways that promote cancer prevention efforts.

## Purpose

The Texas A&M School of Public Health (SPH), located in College Station, Texas, was the focus of the current study. As a Centers for Disease Control and Prevention (CDC)-funded Prevention Research Center and a partner in the Cancer Prevention and Control Research Network, the SPH identified cancer prevention as a priority area in both research and practice. The SPH applied for and attained CEO Cancer Gold Standard accreditation in December 2013, thereby becoming the first school of public health in Texas to earn accreditation.

The purpose of the current study was to assess the extent to which a multifaceted health promotion campaign affected organizational capacity to meet or exceed requirements to become CEO Cancer Gold Standard accredited. The study objectives were to: 1) describe the sample responding to our surveys; 2) document the percent improvement in perceived organizational change across the Five Pillars; and 3) examine whether participant characteristics (i.e. participants’ university status) influenced perceptions of organizational change. The health and wellness strategies implemented at the SPH were designed to target all workplace participants through the influence on normative beliefs and multi-dimensional cues to action. The strategies also took into account elements of the ecological model by providing cues on individual choice and signaling in the physical and social environments, both aspects of the ecological theory.

## Methods

To evaluate the impact of several existing and new health and wellness activities, researchers at Texas A&M SPH used online surveys delivered via a school-wide email directory. The sampling frame of affiliates of the SPH included 681 participants, which consisted of 60 faculty, 79 staff members, and 542 students available at the time of survey distribution.

The survey assessed perceived organizational values and support for cancer prevention and early detection, physical activity, nutrition, and accessing information on cancer clinical trials. Survey questions measured participants’ level of agreement with the following statements: ‘*SPH supports a tobacco-free campus*;’ ‘*SPH has a culture that values, supports and promotes healthy food choices*;’ ‘*SPH has a culture that values, supports and promotes physical activity*;’ ‘*SPH has a culture that values, supports and promotes prevention, screening and early detection of cancer*;’ ‘*SPH has a culture that values, supports and promotes accessing cancer treatment and clinical trials.*’ Responses were measured on a 6-item Likert-type scale ranging from ‘*don’t know,’ ‘strongly agree,’ ‘agree somewhat,’ ‘neutral,’ ‘disagree somewhat,’ ‘strongly disagree*.’

Baseline (response rate = 25 %, *n* = 173) and follow-up (response rate = 21 %, *n* = 148) surveys were emailed six months apart to faculty, staff, and students at the SPH. We analyzed respondents’ surveys at baseline (September, 2013) and follow-up (March, 2014). Approximately, 44 % of follow-up survey respondents also took the baseline survey. Responses were not linked across time; rather, each survey time point served as a snap-shot of current organizational values. There was no requirement or monetary incentive for respondents to take the online survey.

We measured the percent improvement among respondents completing either baseline or follow-up surveys. We used the following formula to calculate the percent improvement among respondents:

$$ Percent\  Improvement=100 \times \frac{\%\ \mathrm{Agreement}\ \mathrm{at}\ \mathrm{Time}2-\%\ \mathrm{Agreement}\ \mathrm{at}\ \mathrm{Time}1}{\%\ \mathrm{Agreement}\ \mathrm{at}\ \mathrm{Time}1}. $$

*Where Time 1 and Time 2 includes the percent of participants in either agreement or disagreement (agree and disagree, calculated separately) out of all survey respondents answering the survey item (excluding responses for neutral or don’t know). For example, if 80 % agree at baseline and 100 % agree at follow-up, the calculation would be (100–80) ÷ 80 × 100 = 25 % improvement*.

We conducted bivariate and multivariate analyses to assess the change from baseline using the binary response of agreement (combining response choices of ‘*strongly agree’* and ‘*agree somewhat’*) [32] and disagreement (combining response choices of ‘*disagree somewhat’* and ‘*strongly disagree’*) as the outcomes across all respondents. Transforming this variable into a dichotomous form enabled us to have large enough cell sizes for positive and negative responses to make meaningful comparisons and simplify interpretations. Responses for don’t know or neutral were excluded from analyses because we were only interested in measuring potential changes amongst those either in agreement or disagreement. In the bivariate descriptive analyses, we presented results in terms of percent improvement in agreement. Chi Square tests were used to determine whether sample characteristics differed from baseline to follow-up. In the bivariate and multivariate logistic regression models, we dichotomized the outcome variable into agreement versus disagreement. Odds ratios were used to identify differences from baseline to follow-up. All multivariate regression analyses controlled for participants’ university status (i.e. faculty, staff, or student). Institutional Review Board (IRB) approval was granted by Texas A&M.

### Multifaceted health & wellness campaign

We tested the impact of a multifaceted health promotion campaign including: monthly digital newsletters promoting wellness and cancer awareness; signage (nutrition and physical activity prompts); encouragement to participate in a statewide web-based physical activity competition; digital media displays with information on how to access cancer clinical trials; and digital messages displaying senior staff encouraging cancer screening and healthy eating. Many of these items were based on principles from the Health Belief Model [33] focusing on cues to action (e.g. emails, signs, digital displays) and the Theory of Reasoned Action and Planned Behavior [34] focusing on normative beliefs (e.g. highlighting senior leaders making healthy choices). For the health promotion activities, these theories were used as a *guide* for the campaign but not fully integrated into all activities. Health and wellness information was taken from a variety of sources including, but not limited to, the CDC, the American Cancer Society (ACS), and the American Heart Association (AHA). Additionally, SPH increased opportunities for engaging in healthy lifestyles by redesigning the workplace in several ways including: making filtered water more accessible, providing healthy food choices for meetings, increasing storage for healthy foods, and promoting standing offices and desks in classroom areas.

## Results

Table 1 provides the distribution of our sample. Overall, the majority of respondents self-identified as being female (74.1 %), White (73.5 %), and non-Hispanic (85.4 %). In addition, most were students, followed by staff, and faculty. There was no significant difference (alpha = 0.05) between baseline and follow-up across sample characteristics.

**Table 1:** Distribution of sample by selected characteristics

		Baseline n (Column %)	Follow-up n (Column %)	Total n (Column %)
Sex	Male	36 (23.4 %)	31 (29.5 %)	67 (25.9 %)
	Female	118 (76.6 %)	74 (70.5 %)	192 (74.1 %)
Race	White	98 (74.2 %)	68 (72.3 %)	166 (73.5 %)
	Black	12 (9.1 %)	9 (9.6 %)	21 (9.3 %)
	Asian	18 (13.6 %)	15 (16.0 %)	33 (14.6 %)
	American Indian or Alaska Native	3 (2.3 %)	2 (2.1 %)	5 (2.2 %)
	Native Hawaiian of Other Pacific Islander	1 (0.8 %)	0 (0 %)	1 (0.4 %)
Ethnicity	Hispanic	22 (14.3 %)	16 (15.1 %)	38 (14.6 %)
	Non-Hispanic	132 (85.7 %)	90 (84.9 %)	222 (85.4 %)
Participants’ University Status	Faculty	30 (19.6 %)	16 (14.8 %)	46 (17.6 %)
	Staff	38 (24.8 %)	26 (24.1 %)	64 (24.5 %)
	Student (full-time)	73 (47.7 %)	59 (54.6 %)	132 (50.6 %)
	Student (part-time)	12 (7.8 %)	7 (6.5 %)	19 (7.3 %)

Percent is calculated with variable grouping [e.g. males at baseline, 36/ (36 + 118) = 23.4 %]

No significant differences were present across sample characteristics from baseline to follow-up using chi sq. (*p* < .05)

Table 2 presents respondents’ level of agreement with items pertaining to a culture that values, supports, and promotes various cancer-prevention items. Approximately 97 % of respondents indicated agreement that ‘*SPH supports a tobacco-free campus,*’ both at baseline and follow-up. With respect to SPH supporting *healthy food choices,* there was 25.0 % disagreement at baseline and 17.7 % disagreement at follow-up, which represents 29.2 % improvement over time. There was 75.0 % agreement at baseline and 82.4 % agreement at follow-up, which represents 9.9 % improvement over time. With respect to *‘physical activity’* there was 17.8 % disagreement at baseline and 18.6 % disagreement at follow-up, which represents 4.5 % decline in percent improvement (−4.5 % improvement) over time. There was 82.2 % agreement at baseline and 81.4 % agreement at follow-up, which represents 1.0 % decline in percent improvement (−1.0 % improvement) over time.

**Table 2:** Distribution of sample by selected outcomes

	Level of agreement^a^	Baseline n (Column %)	Follow-up n (Column %)	Total (Column %)	Percent improvement	P-value*
Tobacco	Disagreed	4 (2.9 %)	3 (2.9 %)	7 (2.9 %)	0 %	0.7055
	Agreed	132 (97.1 %)	101 (97.1 %)	233(97.1 %)	0 %	0.0423
Nutrition	Disagreed	32 (25.0 %)	18 (17.7 %)	50(21.7 %)	29.2 %	0.0477
	Agreed	96 (75.0 %)	84 (82.4 %)	180(78.3 %)	9.9 %	0.3711
Physical activity	Disagreed	21 (17.8 %)	19 (18.6 %)	40(18.2 %)	−4.5 %	0.7518
	Agreed	97 (82.2 %)	83 (81.4 %)	180(81.8 %)	−1.0 %	0.2967
Screening	Disagreed	22 (23.2 %)	8 (9.8 %)	30(17.0 %)	57.8 %	0.0106
	Agreed	73 (76.8 %)	74 (90.2 %)	147(83.1 %)	17.5 %	0.9343
Clinical trials	Disagreed	32 (33.0 %)	9 (13.6 %)	41(25.2 %)	58.8 %	0.0003
	Agreed	65 (67.0 %)	57 (86.4 %)	122(74.9 %)	29.0 %	0.4689

*n* = 180 at baseline and 148 at follow-up. The n varies due to missing data for certain responses

^a^Agreement/Disagreement that SPH valued, supported, and promoted this activity

*chi-square for differences in proportions from baseline to follow-up indicating differences in sample sizes

With respect to *‘prevention, screening* and *early detection of cancer’* there was 23.2 % disagreement at baseline and 9.8 % disagreement at follow-up, which represents 57.8 % improvement over time. There was 76.8 % agreement at baseline and 90.2 % agreement at follow-up, which represents 17.5 % improvement over time. With respect to *‘accessing cancer treatment* and *clinical trials,’* there was 33.0 % disagreement at baseline and 13.6 % disagreement at follow-up, which represents 58.8 % improvement over time. There was 67.0 % agreement at baseline and 86.4 % agreement at follow-up, which represents 29.0 % improvement over time.

### Regression analyses

Table 3 presents results of bivariate logistic regression. Overall, only *‘prevention, screening* and *early detection of cancer’* and *‘accessing cancer treatment* and *clinical trials’* improved (*p* < .05) from baseline to follow-up. Table 4 present results of multivariate logistic regression analyses. Individuals at follow-up were more likely to report agreement with the statements related to *prevention, screening and early detection of cancer* (OR = 3.60, CI = 1.27–10.18) than those at baseline, after controlling for participants’ university status. Similarly, respondents at follow-up were more likely to report agreement with the statement about *accessing cancer treatment and clinical trials* (OR = 3.99, CI = 1.53–10.41) than those at baseline, after controlling for participants’ university status. No significant differences in organizational culture were reported for organizational change related to valuing, supporting, and promoting cancer-related lifestyle behaviors (e.g. physical activity and nutrition).

**Table 3:** Unadjusted analyses: logistic regression predicting change in agreement with organizational value and support from baseline to follow-up

		Odds ratio	95 % confidence intervals	
			Lower	Upper
Tobacco	Follow-up vs. Baseline	1.020	0.223	4.661
Nutrition	Follow-up vs. Baseline	1.555	0.814	2.972
Physical activity	Follow-up vs. Baseline	0.946	0.476	1.879
Screening	Follow-up vs. Baseline	2.788*	1.166	6.663
Clinical trials	Follow-up vs. Baseline	3.118*	1.372	7.083

*n* = 180 at baseline and 148 at follow-up. The n varies due to missing data for certain responses

*Indicates significantly (*p* < .05) different

**Table 4:** Adjusted analyses: logistic regression predicting change in agreement with organizational value and support from baseline to follow-up

		Odds Ratio	95 % confidence intervals	
			Lower	Upper
Tobacco	Follow-up vs. Baseline	#	#	#
	Faculty Vs. Student	#	#	#
	Staff Vs. Student	#	#	#
Nutrition	Follow-up vs. Baseline	1.850	0.896	3.818
	Faculty Vs. Student	1.331	0.523	3.391
	Staff Vs. Student	1.606	0.670	3.848
Physical activity	Follow-up vs. Baseline	1.232	0.573	2.649
	Faculty Vs. Student	1.256	0.431	3.658
	Staff Vs. Student	1.216	0.496	2.981
Screening	Follow-up vs. Baseline	3.595*	1.270	10.177
	Faculty Vs. Student	1.490	0.388	5.725
	Staff Vs. Student	1.234	0.434	3.507
Clinical trials	Follow-up vs. Baseline	3.985*	1.525	10.413
	Faculty Vs. Student	0.888	0.295	2.676
	Staff Vs. Student	2.051	0.742	5.668

Adjusted analyses accounts for participants’ university status

*Indicates significantly (*p* < .05) different

^#^Quasi-complete separation of data points detected. Analyses not shown due to the limited cell size

## Discussion

Health promotion and health education efforts utilizing multiple approaches, including digital media, can be effective at improving perceived organizational values/support. Efforts to encourage more organizations to undertake similar efforts are needed to assess broader impacts. In terms of descriptive analyses using percent improvement, nutrition, cancer prevention and early detection, and accessing information on cancer clinical trials all showed improvement from baseline; however, agreement with nutrition was not significantly different in regression analysis. This lack of significant change for nutrition may be related to the already high proportion agreeing with these measures at baseline (i.e. ceiling effects). Additionally, multivariate findings revealed both perceived organizational values/support for cancer prevention and early detection, and accessing information on cancer clinical trials improved dramatically. Improvement across prevention, screening and early detection of cancer, and accessing cancer treatment and clinical trials, is promising given these are strongly tied to improvements in cancer prevention and treatment [35, 36]. There was no difference across participants’ university status, which may indicate individuals were reached equally, regardless of being faculty, staff, or student members. This may also indicate that the messaging and interventions used were universally impactful on the worksite culture.

A recent review found evidence to support the use of the worksite as a setting to support improvements among employees for healthier diets and increased physical activity using various strategies (e.g. cues to action or prompts, food or beverage labeling/information, environmental modifications including worksite fitness centers) [37]. The American Heart Association (AHA) recommends a combination of approaches (e.g. tobacco prevention, physical activity promotion, early detection, and screening) [38]. The multi-component focus of the CEO Cancer Gold Standard accreditation aligns well with such an approach.

Thus, we recommend further study of this multifaceted-component approach. The individual aspects of this multifaceted-component approach should be developed in reference to a theoretical foundation, while being tailored to mechanisms (e.g. digital media, email, and signage) that best meet with the needs, resources, and target audience of one’s organization. Successful health promotion strategies will likely differ by organization to some extent, but the overall method of using a multifaceted-component approach is recommended.

### Limitations

There were several limitations in this study. First, the limited sample size may be a contributing factor to the inability to detect significant improvements over time for behavioral measures (i.e. tobacco, nutrition, physical activity). Additionally, the sample size was limited for analysis across time, preventing multivariate analyses with multiple covariates. Thus, we restricted analyses to only one control variable (participants’ university status). However, our analyses describe change across time and for the SPH as a whole. While the identification of changes for certain groups (e.g. race, sex) was not possible, we were able to describe the overall organizational change. In addition, our ability to further interpret and contextualize improvements over time was limited because information on socioeconomic status, insurance status, or disease status was not included in the current analyses. Further, seasonality effects were not assessed, which may have influenced participants’ engagement in physical activity (frequency, duration, and activity type). While we were able to identify the month in which individuals completed the survey, daily temperatures were not measured. Additionally, changes over time could be attributed to students relocating to attend school in the Fall semester, which may influence their health-related activities based on their awareness of available services/resources, changes in insurance status, or access to healthcare services. Similarly, our response rate was less than 25 % which, while low, is not completely unusual in survey research [39]. Further, we were unable to determine whether respondents were systematically different when compared to non-respondents. Respondents may have represented a proportion of the organization who were more aware of factors contributing to their individual health. As reported in the 2015 profile of students reported on the SPH website (data not shown), 58 % of students were female, where as 72 % of student respondents in our analyses were female [40]. However, approximately 20 % of student respondents (data not shown) reported being Hispanic versus 22 % reported in the 2015 profile of students [40]. Data on faculty and staff profiles were not made available for the current comparisons between our sample and the entire SPH population, which is why we only focused on sample versus population comparisons here among students. Thus, those responding to the survey may not closely mirror the actual SPH population. Consequently, self-selection bias may have limited the generalizability of survey respondents’ responses to the entire organization population.

## Conclusions

The implications associated with worksite wellness policies and worksite culture and values are potentially far-reaching. The implications should be interpreted in light of the relatively small sample size and potential for self-selection bias along with other limitations presented earlier. The societal cost of absenteeism, presenteeism, and the medical costs associated with a workforce that is exposed to potentially avoidable health issues is immense [41–43]. Identifying policies that can be implemented to reduce or eliminate potentially avoidable costs is critical. An example is the CEO accreditation requirement to implement a tobacco-free workplace which supports the Healthy People 2020 goal to increase the percentage of adult (over 18) employees covered by a worksite policy prohibiting smoking from 75.3 to 100 % by 2020 [44]. More research is needed to determine if similar policies, such as the implementation of the CEO Cancer Gold Standard accreditation and persistence of such policies over time, can reduce healthcare costs for both employees and employers. The present analyses focused on organizational values, not cost. However, it is a step in the right direction when it comes to identifying the extent to which worksite initiatives can impact factors necessary for embedding and sustaining worksite initiatives with the potential for both employer and employee benefit.
